# Rhythm-specific regional variation in neurological outcomes after out-of-hospital cardiac arrest: a nationwide registry study in Japan

**DOI:** 10.1016/j.resplu.2026.101514

**Published:** 2026-10-06

**Authors:** Kota Shinada, Yuichiro Sakamoto, Ayaka Matsuoka, Hiroyuki Koami

**Affiliations:** Department of Emergency and Critical Care Medicine, Faculty of Medicine, Saga University, 5-1-1 Nabeshima, Saga City, Saga 849-8501, Japan

**Keywords:** Asystole, Geographic variation, Emergency medical services, Neurological outcome, Mixed-effects model

## Abstract

**Background:**

Regional variation in outcomes after out-of-hospital cardiac arrest (OHCA) has been reported worldwide; however, whether its magnitude differs by initial cardiac rhythm remains unknown. This study aimed to evaluate prefecture-level variation in neurological outcomes after OHCA according to initial cardiac rhythm.

**Methods:**

This retrospective observational study included adult patients with atraumatic OHCA registered in the All-Japan Utstein Registry from 2019 to 2023. Patients were categorized as presenting with a shockable rhythm, pulseless electrical activity (PEA), or asystole according to the initial cardiac rhythm. Mixed-effects logistic regression models were fitted separately for each rhythm group, with favorable neurological outcome at 1 month (defined as a Cerebral Performance Category of 1–2) as the dependent variable and prefecture as a random effect. Prefecture-level variation was quantified using the median odds ratios (MORs).

**Results:**

Among 616,519 participants, 46,575 had a shockable rhythm, 142,073 had PEA, and 427,871 had asystole. Favorable neurological outcomes occurred in 22.3%, 2.7%, and 0.2% of patients, respectively. The MORs were 1.19 for shockable rhythm, 1.31 for PEA, and 1.65 for asystole, indicating progressively greater prefecture-level variation across rhythm categories.

**Conclusions:**

The magnitude of prefecture-level variation in favorable neurological outcomes differed by initial cardiac rhythm categories and was greatest among patients with asystole. The mechanisms underlying these regional differences remain unclear. Future investigations of regional and healthcare system characteristics may help identify potentially modifiable factors associated with disparities in OHCA outcomes.

## Introduction

Out-of-hospital cardiac arrest (OHCA) remains a major global public health challenge and is associated with high mortality despite advances in resuscitation care.^1^ Improving outcomes after OHCA requires not only optimizing individual patient management but also assessing the performance of emergency medical services (EMS) and regional healthcare systems.^2^, ^3^, ^4^

Previous studies have demonstrated substantial geographical variations in OHCA outcomes across countries, regions, and healthcare systems.^1^, ^5^, ^6^, ^7^, ^8^, ^9^ These variations may reflect differences in bystander response, EMS performance, hospital resources, and regional resuscitation practices.^6^, ^9^, ^10^ In Japan, several studies using the nationwide Utstein Registry have documented regional variation in OHCA outcomes.^11^, ^12^, ^13^ However, whether the magnitude of this variation differs by initial cardiac rhythm remains unclear.

Initial cardiac rhythm is a major determinant of outcomes after OHCA. Patients presenting with a shockable rhythm have substantially better outcomes compared with those presenting with pulseless electrical activity (PEA) or asystole.^14^, ^15^ The prognosis and potential for successful resuscitation differ markedly by initial cardiac rhythm, suggesting that regional variation in outcomes may also differ across rhythm categories. Patients presenting with asystole have the poorest prognosis and may be particularly susceptible to regional differences in early cardiac arrest recognition, bystander and EMS response, resuscitation practices, transport systems, and subsequent hospital care. As favorable neurological outcomes after asystole are rare, even modest differences in these factors may produce substantial relative differences in outcomes across regions. However, whether the magnitude of geographic variation in post-OHCA outcomes differs by initial cardiac rhythm has not been systematically investigated.

Using data from a nationwide Utstein registry, this study aimed to evaluate prefecture-level variations in post-OHCA neurological outcomes by initial cardiac rhythm. The magnitude of regional variation was hypothesized to differ across rhythm categories, with the greatest variation expected among patients presenting with asystole.

## Methods

### Study design and participants

This retrospective observational study used data from the All-Japan Utstein Registry collected between 2019 and 2023. The registry systematically records standardized OHCA data in accordance with the Utstein reporting framework.^16^ The analysis included adult patients with atraumatic OHCA whose initial cardiac rhythm was classified as shockable, PEA, or asystole based on bystander automated external defibrillator (AED) information or the initial rhythm recorded by EMS. Patients younger than 18 years of age, those with traumatic OHCA, and those with implausible time intervals were excluded. Implausible intervals were defined as negative values or values exceeding 24 h between the emergency call and EMS contact or between EMS contact and hospital arrival. EMS resuscitation attempts were not an eligibility criterion.

This study was approved by the Institutional Review Board of the Saga University Hospital (approval number: 2026-04-R-01). The registry data were fully anonymized, and the requirement for informed consent was waived. This study was conducted in accordance with institutional policies and the principles outlined in the Declaration of Helsinki.

### Study setting and prehospital treatment for OHCA in Japan

According to population estimates from the Statistics Bureau of Japan, the national population declined from 126.55 million on October 1, 2019, to 124.35 million on October 1, 2023. Japan comprises 47 prefectures with diverse geographic and demographic characteristics.

EMS personnel provide resuscitation care in accordance with the Japan Resuscitation Council (JRC) guidelines,^17^, ^18^ which are based on recommendations from the International Liaison Committee on Resuscitation and adapted to Japanese protocols. Specially trained EMS personnel are authorized to perform advanced airway management and administer adrenaline under EMS medical oversight. This role differs from “physician-provided advanced life support (ALS)” recorded in the registry, which specifically refers to prehospital resuscitation procedures performed directly by a physician. Direct physician involvement in prehospital care may also occur through physician dispatch programs.

Although Japan has a nationally established framework for prehospital emergency care, EMS systems are organized and operated locally. Accordingly, regional differences may exist in call-taking and dispatch procedures, EMS resource deployment, physician-staffed prehospital care, transport strategies, and access to receiving hospitals. However, the registry lacked detailed prefecture-level data on these characteristics. Therefore, this study was not designed to identify specific regional system factors underlying the observed variation in outcomes.

### Data collection and quality control

Data were prospectively collected using standardized Utstein-style data forms. Variables included demographic characteristics, arrest circumstances, prehospital resuscitation interventions, time intervals, and outcomes. EMS personnel and receiving hospitals completed forms, which were subsequently compiled by the Fire and Disaster Management Agency.

In accordance with the 2024 Utstein reporting recommendations,^16^ bystander AED information was incorporated into the initial rhythm classification. As AED use coding changed during the study period, use was recorded as “checked” or “not checked” in 2019–2020, with “checked” indicating bystander defibrillation, and as “shock indicated and delivered,” “shock indicated but not delivered,” “shock not indicated,” or “unknown” from 2021 onward. To harmonize definitions across the study period, patients classified as “checked” in 2019–2020 or as having “shock indicated and delivered” or “shock indicated but not delivered” from 2021 onward were classified as having a shockable rhythm, regardless of the initial rhythm subsequently recorded by EMS. For all other patients, including those without available AED information, rhythm classification was based on the initial rhythm recorded by EMS.

For prehospital advanced airway use and adrenaline administration, records without documentation of an intervention, including those with missing information, were coded as indicating that the intervention was not performed, consistent with the registry coding structure. Patients with implausible time intervals, defined as negative values or intervals exceeding 24 h between the emergency call and EMS contact or between EMS contact and hospital arrival, were excluded. No additional exclusions were made for logical inconsistencies in other variables.

### Outcome

The primary endpoint was a favorable neurological outcome at 1 month, defined as 1-month survival with a Cerebral Performance Category (CPC) score of 1 or 2.

### Statistical analysis

Analyses were performed separately for patients with a shockable rhythm, PEA, and asystole to assess prefecture-level variation in neurological outcomes by initial cardiac rhythm. The primary outcome was a favorable neurological outcome, defined as 1-month survival with a CPC score of 1–2. For each rhythm category, a mixed-effects logistic regression model was fitted with prefecture as a random effect.

The primary model included age, sex, physician-provided prehospital advanced life support, witnessed arrest, bystander cardiopulmonary resuscitation (CPR), cardiac etiology, time from emergency call to EMS contact, and time from EMS contact to hospital arrival as fixed effects. Prehospital adrenaline administration and advanced airway management were excluded, as these interventions occur after EMS contact and may reflect regional differences in prehospital system performance. Bystander AED shock was used to classify the initial rhythm and was, therefore, not included as an adjustment variable.

Prefecture-level variation was quantified using the variance of the prefecture-level random effects and the median odds ratio (MOR), which represents the median increase in the odds of a favorable outcome when comparing otherwise identical patients from two randomly selected prefectures. MORs and 95% confidence intervals (CIs) were estimated for each rhythm category. Prefecture-specific random-effects odds ratios (ORs) and conditional 95% CIs were also estimated to characterize regional outcome patterns.

To assess whether prefecture-level variations differed across rhythm categories, an additional mixed-effects logistic regression model was fitted across all three rhythm categories, with separate prefecture-level random-effect variances specified for each category. This model was compared with a model assuming a common prefecture-level variance across categories using a likelihood-ratio test. Pairwise comparisons were performed by constraining the prefecture-level variances of each pair of rhythm categories to equality and comparing the constrained and unconstrained models using likelihood-ratio tests. *P*-values from the three pairwise comparisons were adjusted using the Holm method.

Sensitivity analyses were conducted to assess the robustness of the primary findings. First, prehospital adrenaline administration and advanced airway management were included as fixed effects. Second, bystander CPR was excluded, as it may reflect community- and system-level performance.

In the exploratory subgroup analysis, patients with asystole were stratified by witnessed arrest status. Separate mixed-effects logistic regression models were fitted using the same covariates as in the primary model, and MORs with 95% CIs were estimated for each subgroup. To formally compare prefecture-level variation between witnessed and unwitnessed arrests, a combined model allowing separate prefecture-level random-effect variances according to witnessed arrest status was compared with a model assuming a common variance using a likelihood-ratio test.

To assess the similarity of prefecture-level effects across rhythm categories, Pearson’s correlation coefficients with 95% CIs were calculated between prefecture-specific random effects on the log-odds scale obtained from the rhythm-specific mixed-effects models.

All statistical analyses were performed using JMP Student Edition 19 (SAS Institute, Cary, NC, USA) and R version 4.6.0 (R Foundation for Statistical Computing, Vienna, Austria). All statistical tests were two-sided, and a *P* value of <0.05 was considered significant.

## Results

### Study population and baseline characteristics

Between 2019 and 2023, 664,646 patients with OHCA were registered in the All-Japan Utstein Registry. After applying the inclusion and exclusion criteria, only 616,519 patients were included in the analysis. Among these, 46,575 had a shockable rhythm, 142,073 had PEA, and 427,871 had asystole (Fig. 1).

**Fig. 1:**
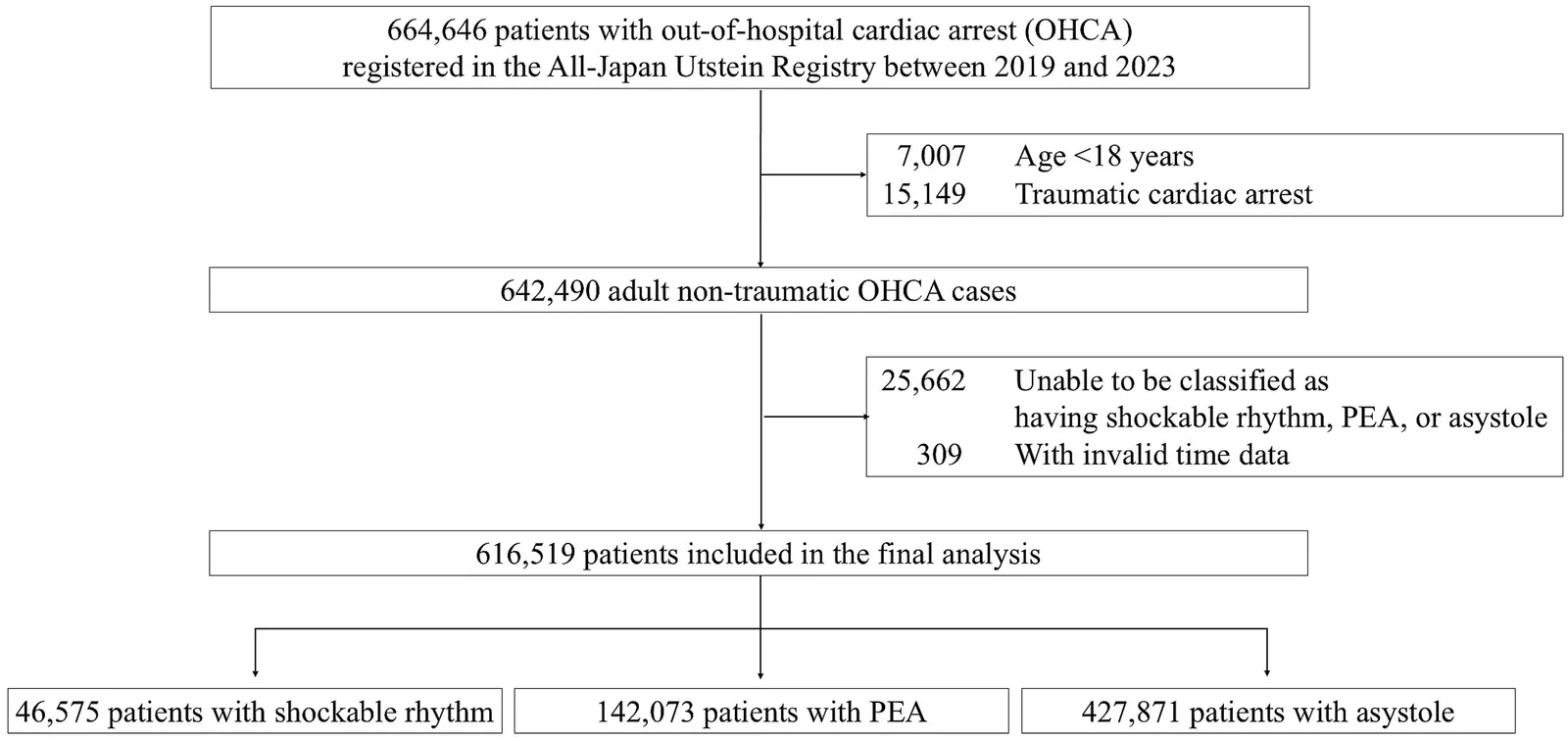
**Flow diagram illustrating the patient selection process**.

Bystander AED information was missing for 386,236 patients, whereas information on prehospital advanced airway use and adrenaline administration was missing for 35,330 and 31,262 patients, respectively.

The baseline characteristics by initial cardiac rhythm are presented in Table 1. Patients with a shockable rhythm were younger, predominantly men, and more likely to have a cardiac etiology than those with PEA or asystole. Shockable rhythm and PEA were more common than asystole. Favorable neurological outcomes at 1 month occurred in 22.3%, 2.7%, and 0.2% of patients with shockable rhythm, PEA, and asystole, respectively.

**Table 1:** Baseline characteristics stratified by cardiac rhythm.

	**Shockable** **(*n* = 46,575)**	**PEA** **(*n* = 142,073)**	**Asystole** **(*n* = 427,871)**
Age, y/o	70 [58–80]	81 [72–88]	81 [72–88]
Sex (male)	35,393 (76.0%)	82,416 (58.0%)	235,395 (55.0%)
Etiology (cardiac)	41,637 (89.4%)	87,978 (61.9%)	275,052 (64.3%)
Witnessed	33,457 (71.8%)	99,241 (69.9%)	106,069 (24.8%)
Bystander CPR	29,945 (64.3%)	60,077 (42.3%)	231,871 (54.2%)
Prehospital advanced airway usage	19,996 (42.9%)	68,067 (47.9%)	187,234 (43.8%)
Prehospital adrenaline administration	15,947 (34.2%)	52,883 (37.2%)	110,893 (25.9%)
Prehospital physician-provided ALS	5643 (12.1%)	12,888 (9.1%)	30,185 (7.1%)
Call-to-EMS contact time, minute	9 [7–11]	9 [7–11]	9 [8–12]
EMS contact-to-hospital arrival time, minute	24 [18–31]	24 [19–31]	24 [18–31]
One-month survival with a CPC score of 1–2	10,371 (22.3%)	3853 (2.7%)	837 (0.2%)

Continuous variables were expressed as medians (interquartile ranges), whereas categorical variables were expressed as counts (percentages). Comparisons between the rhythm groups were conducted for descriptive purposes only, without formal hypothesis testing.

**Abbreviations**: ALS, advanced life support; CPC, Cerebral Performance Category; CPR, cardiopulmonary resuscitation; EMS, emergency medical services; PEA, pulseless electrical activity.

Crude prefecture-level rates of favorable neurological outcomes and the use of prehospital advanced airway management and adrenaline varied across all rhythm categories (Supplementary Table 1). The ranges of favorable neurological outcome rates were 12.8–28.6% for shockable rhythm, 1.1–6.1% for PEA, and 0.04–1.10% for asystole.

### Prefecture-level variation by rhythm category

In the primary mixed-effects logistic regression models, the magnitude of prefecture-level variation in favorable neurological outcomes differed by initial cardiac rhythm. The MORs were 1.19 (95% CI, 1.14–1.26) for shockable rhythm, 1.31 (95% CI, 1.23–1.42) for PEA, and 1.65 (95% CI, 1.47–1.91) for asystole (Table 2).

**Table 2:** Prefecture-level variation in favorable neurological outcomes by cardiac rhythm.

	**Shockable MOR (95% CI)**	**PEA MOR (95% CI)**	**Asystole MOR (95% CI)**
Primary model	1.19 (1.14–1.26)	1.31 (1.23–1.42)	1.65 (1.47–1.91)
Additional adjustment for prehospital advanced airway usage and adrenaline administration	1.38 (1.29–1.49)	1.35 (1.26–1.46)	1.65 (1.47–1.91)
Primary model excluding bystander CPR	1.20 (1.15–1.26)	1.30 (1.22–1.40)	1.63 (1.45–1.89)

Prefecture-level variation in favorable neurological outcomes was evaluated using mixed-effects logistic regression models, with prefecture included as a random effect. Median odds ratios (MORs) and 95% confidence intervals were calculated for each rhythm category. Higher MORs indicated greater between-prefecture variation in the odds of favorable neurological outcomes. The primary model was adjusted for age, sex, physician-provided advanced life support in the prehospital setting, witnessed arrest, bystander cardiopulmonary resuscitation (CPR), cardiac etiology, time from emergency call to EMS contact, and time from EMS contact to hospital arrival. Sensitivity analyses included additional adjustments for prehospital advanced airway use and adrenaline administration as well as the exclusion of bystander CPR from the adjustment variables.

**Abbreviations:** CI, confidence interval; CPR, cardiopulmonary resuscitation; EMS, emergency medical services; MOR, median odds ratio; PEA, pulseless electrical activity.

An additional model formally comparing prefecture-level variances across rhythm categories showed a significant overall difference (likelihood ratio test, *P* < 0.001). Pairwise comparisons demonstrated significant differences between shockable rhythm and PEA (Holm-adjusted *P* = 0.016), between shockable rhythm and asystole (Holm-adjusted *P* < 0.001), and between PEA and asystole (Holm-adjusted *P* = 0.004).

Prefecture-specific random-effects ORs and conditional 95% CIs are shown in Fig. 2 and Supplementary Table 2. The ORs ranged from 0.62 to 1.36 for shockable rhythm, 0.57 to 2.06 for PEA, and 0.39 to 4.66 for asystole. Prefecture-specific random effects were positively correlated across rhythm categories. Pearson’s correlation coefficients were 0.74 (95% CI, 0.57–0.85) between shockable rhythm and PEA, 0.54 (95% CI, 0.30–0.72) between shockable rhythm and asystole, and 0.79 (95% CI, 0.65–0.88) between PEA and asystole.

**Fig. 2:**
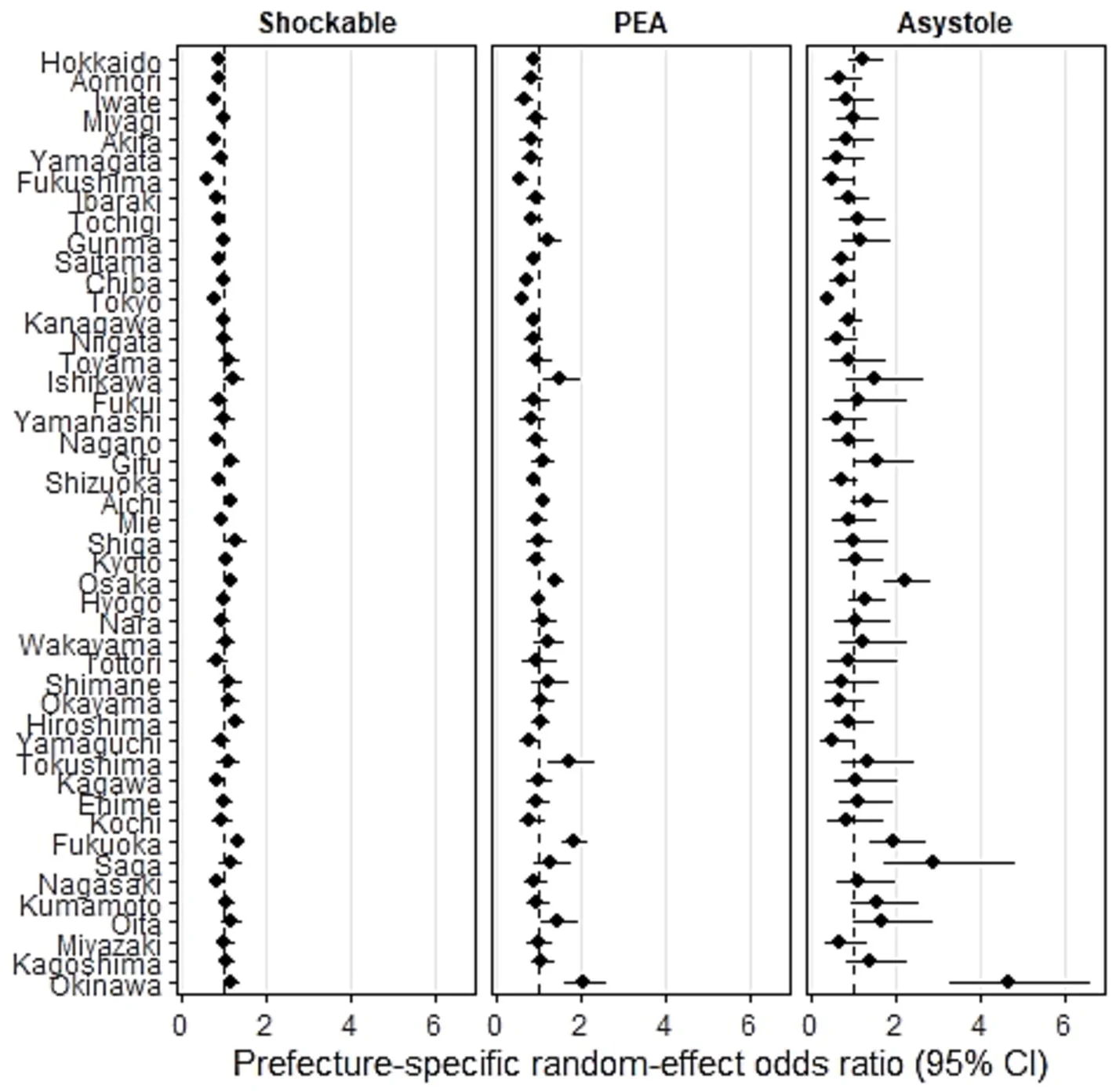
**Prefecture-specific random-effect odds ratios for favorable neurological outcomes by cardiac rhythm**.

### Sensitivity analyses

The pattern of prefecture-level variation remained generally consistent across the sensitivity analyses (Table 2). After additional adjustment for prehospital advanced airway management and adrenaline administration, the MORs were 1.38 (95% CI, 1.29–1.49) for shockable rhythm, 1.35 (95% CI, 1.26–1.46) for PEA, and 1.65 (95% CI, 1.47–1.91) for asystole. When bystander CPR was excluded from the adjustment variables, the corresponding MORs were 1.20 (95% CI, 1.15–1.26), 1.30 (95% CI, 1.22–1.40), and 1.63 (95% CI, 1.45–1.89), respectively. In both sensitivity analyses, the MOR was highest among patients with asystole.

### Exploratory analysis according to witnessed status among patients with asystole

In the exploratory subgroup analysis, prefecture-level variation was observed for both witnessed and unwitnessed cardiac arrests among patients with asystole. The MORs were 1.63 (95% CI, 1.44–1.92) for witnessed arrests and 1.97 (95% CI, 1.64–2.54) for unwitnessed arrests. Although the MOR was numerically higher for unwitnessed arrests, the difference in prefecture-level variance between the two groups was not significant (likelihood ratio test, *P* = 0.071).

## Discussion

In this nationwide registry study, prefecture-level variation in neurological outcomes after OHCA differed by initial cardiac rhythm. The MORs were 1.19 (95% CI, 1.14–1.26), 1.31 (95% CI, 1.23–1.42), and 1.65 (95% CI, 1.47–1.91) for shockable rhythm, PEA, and asystole, respectively. Formal comparisons of prefecture-level variances confirmed significant differences between all three rhythm categories. The MOR remained highest among patients with asystole in sensitivity analyses that additionally adjusted for prehospital advanced airway management and adrenaline administration or excluded bystander CPR from the adjustment variables. The MOR remained consistently highest among patients with asystole. Furthermore, prefecture-specific random effects were positively correlated across rhythm categories. In an exploratory analysis restricted to patients with asystole, prefecture-level variation was observed for both witnessed and unwitnessed arrests. Although the MOR was numerically higher for unwitnessed arrests, the difference in prefecture-level variance was not significant. Overall, these findings support rhythm-specific differences in the magnitude of prefecture-level variation in neurological outcomes after OHCA, with the greatest variation observed among patients with asystole.

Patients with a shockable rhythm generally represent a relatively favorable subgroup in which early recognition, bystander CPR, and prompt defibrillation are associated with improved outcomes.^19^, ^20^ By contrast, asystole is associated with a poor prognosis, and favorable neurological outcomes are exceedingly rare.^21^, ^22^ One possible explanation for the greater prefecture-level variation among patients with asystole is that outcomes in this group may be more sensitive to regional differences in the circumstances and systems surrounding resuscitation. These factors may include the timing of cardiac arrest recognition, bystander response, EMS practice, transportation systems, and subsequent hospital care. However, the present study did not directly measure many of these regional characteristics and, therefore, cannot establish whether the greater variation observed among patients with asystole reflects a stronger causal influence of regional systems of care. Alternative explanations should also be considered. As favorable neurological outcomes are extremely uncommon among patients with asystole, small absolute differences in outcome rates across prefectures may translate into larger relative differences. In addition, patients presenting with asystole constitute a clinically heterogeneous population with respect to arrest circumstances, underlying etiology, duration of untreated cardiac arrest, and subsequent treatment decisions. Differences in hospital-level resources, post-resuscitation care, decisions regarding the continuation or termination of resuscitation, and do-not-attempt-resuscitation practices may also contribute to regional differences in outcomes. However, these factors were not fully captured in the registry. Consequently, unmeasured differences in these characteristics across prefectures may have contributed to the greater variation observed in this group.

In the exploratory analysis of patients with asystole, the MOR was numerically higher for unwitnessed than witnessed arrests. Unwitnessed cardiac arrest is more likely to involve delayed recognition and initiation of resuscitation efforts.^23^, ^24^ Consequently, outcomes after an unwitnessed arrest may be influenced by a broader range of community-level characteristics, including public awareness of resuscitation, the prevalence of older adults living alone, response systems in long-term care facilities, and patterns of emergency activation. By contrast, witnessed arrests involve earlier recognition and activation of the emergency care system, which may limit the extent to which such factors contribute to variation in outcomes. However, the difference in prefecture-level variance between witnessed and unwitnessed arrests was not significant (*P* = 0.071). Therefore, this finding does not provide evidence of a systematic difference in prefecture-level variation according to witnessed status and should be interpreted as hypothesis-generating.

Prefecture-specific random effects showed moderate-to-strong positive correlations across rhythm categories. Prefectures with relatively favorable outcomes for one rhythm category tended to have relatively favorable outcomes for the other rhythm categories. This pattern may reflect regional characteristics that influence multiple stages of the OHCA care pathway; however, the specific factors underlying these correlations could not be identified in the present study. Notably, the magnitude of prefecture-level variation differed significantly across rhythm categories despite these positive correlations, suggesting that common regional factors may coexist with rhythm-specific differences in the extent of outcome variation.

Although this study was not designed to identify the specific causes of prefecture-level variation, the findings provide a framework for future investigations of regional disparities in post-OHCA outcomes. The greater variation observed among patients with asystole identifies this population as an important group for further investigation, without establishing a greater causal influence of regional systems of care in this group. Future studies incorporating detailed information on EMS organization, community response, hospital characteristics, and post-resuscitation care and comparisons of regions with different outcome patterns may help identify modifiable factors associated with regional disparities in OHCA outcomes.

This study has some limitations. First, the observational design precludes causal inference, and the factors underlying prefecture-level variation could not be identified directly. Residual confounding from unmeasured regional characteristics, including socioeconomic, geographic, and demographic factors, may have contributed to the observed differences in outcomes. Second, prehospital adrenaline administration and advanced airway management were not included as adjustment variables in the primary analysis, as these interventions may reflect regional system performance. Differences in adrenaline administration may be particularly relevant when comparing rhythm categories, as adrenaline is prioritized in the treatment of non-shockable rhythms. Although additional adjustment for adrenaline administration and advanced airway management in the sensitivity analysis did not materially attenuate the observed prefecture-level variation, residual confounding factors related to differences in prehospital resuscitation practices cannot be excluded. Third, the rarity of favorable neurological outcomes among patients with asystole may have limited the precision of prefecture-specific random-effect estimates. Although mixed-effects modeling shrinks these estimates toward the overall mean, substantial uncertainty remains when the number of favorable outcomes within individual prefectures is small. Moreover, as favorable neurological outcomes were uncommon in this group, small absolute differences in outcome rates across prefectures may translate into larger relative differences. Therefore, both the magnitude of prefecture-level variation and prefecture-specific estimates among patients with asystole should be interpreted with caution. Fourth, prefecture-level effects were aggregated and therefore did not capture variation within prefectures or between hospitals. Information on hospital characteristics, including level of care, specialized post-arrest management, and destination selection, was unavailable. Additionally, the registry did not fully capture differences in the circumstances surrounding resuscitation, post-resuscitation care, or subsequent treatment decisions. These unmeasured factors may have contributed to the observed prefecture-level variation, particularly among patients with asystole. Fifth, the study period encompassed the coronavirus disease 2019 pandemic, during which OHCA care practices and healthcare system capacity may have changed over time and varied across regions. The analysis did not explicitly account for temporal or region-specific effects of the pandemic. These factors may have contributed to the observed prefecture-level variations. Finally, the findings may not be generalizable to EMS systems in other countries.

## Conclusion

Using a nationwide OHCA registry, this study demonstrated that prefecture-level variation in favorable neurological outcomes differed across cardiac rhythm categories and was greatest among patients with asystole. Among patients with asystole, the point estimate of the MOR was numerically higher for unwitnessed than witnessed arrests, although the difference was not significant. These findings indicate rhythm-specific differences in the magnitude of regional variation but do not establish the mechanisms underlying these differences. Future studies comparing regions with different outcome patterns, including high-performing regions, and incorporating detailed regional and healthcare system characteristics may help identify potentially modifiable factors associated with disparities in post-OHCA outcomes.

## Declaration of generative AI and AI-assisted technologies in the manuscript preparation process

During manuscript preparation, ChatGPT (OpenAI) was used to improve the clarity and readability of the text. The authors reviewed and edited the generated content as necessary and assumed full responsibility for the content of this manuscript.

## CRediT authorship contribution statement

**Kota Shinada:** Writing – review & editing, Writing – original draft, Visualization, Validation, Software, Resources, Project administration, Methodology, Investigation, Formal analysis, Data curation, Conceptualization. **Yuichiro Sakamoto:** Writing – review & editing, Supervision. **Ayaka Matsuoka:** Writing – review & editing. **Hiroyuki Koami:** Writing – review & editing.

## Funding

This research did not receive any specific grants from funding agencies in the public, commercial, or nonprofit sectors.

## Declaration of competing interest

The authors declare that they have no known competing financial interests or personal relationships that could have appeared to influence the work reported in this paper.

## Appendix A Supplementary material

Supplementary Data 1 Prefecture-level ranges of neurological outcomes and prehospital interventions according to cardiac rhythm and prefecture-specific random-effect odds ratios.
