# Supplementary material for: Rhythm-specific regional variation in neurological outcomes after out-of-hospital cardiac arrest: a nationwide registry study in Japan
Source: Resusc Plus. 2026 Oct 6;32:101514. doi: 10.1016/j.resplu.2026.101514 (PMC13695374; doi:10.1016/j.resplu.2026.101514)
Supplement: Supplementary file 1 [file mmc1.docx]

**Supplementary Table 1.** Ranges of prefecture-level outcomes and prehospital intervention rates by cardiac rhythm

| Rhythm | Favorable neurological outcome | Prehospital advanced airway usage | Prehospital adrenaline administration |
| --- | --- | --- | --- |
| Shockable | 12.8%–28.6% | 6.6%–67.9% | 8.9%–59.2% |
| PEA | 1.1%–6.1% | 7.7%–73.7% | 16.8%–60.1% |
| Asystole | 0.04%–1.10% | 5.8%–74.0% | 4.6%–59.1% |

Values represent the ranges of crude prefecture-level proportions across the 47 prefectures for favorable neurological outcomes at 1 month, prehospital advanced airway management, and adrenaline administration within each cardiac rhythm category. A favorable neurological outcome was defined as a Cerebral Performance Category score of 1 or 2 at 1 month.

**Abbreviation:** PEA, pulseless electrical activity

**Supplementary Table 2.** Prefecture-specific random-effect odds ratios by cardiac rhythm

| Prefecture | Shockable | PEA | Asystole |
| --- | --- | --- | --- |
| Hokkaido | 0.91 (0.81–1.02) | 0.88 (0.73–1.05) | 1.23 (0.88–1.71) |
| Aomori | 0.89 (0.73–1.08) | 0.85 (0.64–1.13) | 0.69 (0.37–1.26) |
| Iwate | 0.79 (0.64–0.96) | 0.66 (0.48–0.92) | 0.84 (0.46–1.53) |
| Miyagi | 1.02 (0.87–1.19) | 0.97 (0.76–1.23) | 1.03 (0.64–1.64) |
| Akita | 0.79 (0.63–0.98) | 0.82 (0.59–1.14) | 0.82 (0.44–1.53) |
| Yamagata | 0.93 (0.76–1.15) | 0.84 (0.61–1.14) | 0.64 (0.32–1.29) |
| Fukushima | 0.62 (0.51–0.75) | 0.57 (0.42–0.77) | 0.54 (0.29–1.03) |
| Ibaraki | 0.85 (0.73–0.99) | 0.95 (0.76–1.18) | 0.89 (0.56–1.40) |
| Tochigi | 0.91 (0.76–1.07) | 0.87 (0.68–1.11) | 1.12 (0.69–1.81) |
| Gunma | 1.01 (0.86–1.18) | 1.24 (1.00–1.55) | 1.19 (0.75–1.89) |
| Saitama | 0.89 (0.80–1.00) | 0.92 (0.79–1.08) | 0.73 (0.51–1.04) |
| Chiba | 1.00 (0.90–1.12) | 0.73 (0.61–0.88) | 0.71 (0.48–1.04) |
| Tokyo | 0.78 (0.71–0.86) | 0.62 (0.53–0.72) | 0.39 (0.28–0.54) |
| Kanagawa | 1.04 (0.94–1.15) | 0.93 (0.80–1.08) | 0.91 (0.66–1.26) |
| Niigata | 1.04 (0.89–1.22) | 0.90 (0.70–1.14) | 0.64 (0.37–1.12) |
| Toyama | 1.14 (0.92–1.41) | 0.98 (0.73–1.33) | 0.91 (0.47–1.76) |
| Ishikawa | 1.23 (1.00–1.51) | 1.51 (1.14–1.99) | 1.52 (0.87–2.68) |
| Fukui | 0.88 (0.68–1.13) | 0.92 (0.65–1.31) | 1.13 (0.56–2.26) |
| Yamanashi | 1.01 (0.80–1.28) | 0.83 (0.59–1.15) | 0.63 (0.30–1.32) |
| Nagano | 0.84 (0.70–1.01) | 0.95 (0.74–1.21) | 0.91 (0.54–1.53) |
| Gifu | 1.20 (1.02–1.42) | 1.10 (0.87–1.40) | 1.56 (1.00–2.44) |
| Shizuoka | 0.91 (0.79–1.05) | 0.88 (0.72–1.07) | 0.72 (0.45–1.15) |
| Aichi | 1.16 (1.04–1.29) | 1.10 (0.94–1.28) | 1.35 (0.98–1.86) |
| Mie | 0.97 (0.81–1.15) | 0.97 (0.76–1.24) | 0.91 (0.53–1.57) |
| Shiga | 1.32 (1.09–1.59) | 1.00 (0.75–1.33) | 1.02 (0.57–1.84) |
| Kyoto | 1.05 (0.90–1.23) | 0.94 (0.75–1.17) | 1.08 (0.67–1.74) |
| Osaka | 1.16 (1.05–1.28) | 1.40 (1.22–1.60) | 2.21 (1.73–2.84) |
| Hyogo | 0.99 (0.88–1.13) | 0.99 (0.83–1.18) | 1.28 (0.91–1.80) |
| Nara | 0.98 (0.80–1.19) | 1.11 (0.85–1.46) | 1.05 (0.58–1.90) |
| Wakayama | 1.05 (0.85–1.30) | 1.21 (0.91–1.61) | 1.24 (0.67–2.28) |
| Tottori | 0.84 (0.62–1.13) | 0.97 (0.64–1.46) | 0.92 (0.41–2.07) |
| Shimane | 1.13 (0.89–1.45) | 1.21 (0.85–1.73) | 0.75 (0.35–1.62) |
| Okayama | 1.14 (0.95–1.38) | 1.07 (0.83–1.38) | 0.67 (0.36–1.26) |
| Hiroshima | 1.31 (1.13–1.53) | 1.06 (0.86–1.31) | 0.91 (0.55–1.49) |
| Yamaguchi | 0.94 (0.76–1.16) | 0.81 (0.59–1.09) | 0.51 (0.25–1.04) |
| Tokushima | 1.11 (0.88–1.41) | 1.71 (1.25–2.32) | 1.33 (0.71–2.46) |
| Kagawa | 0.85 (0.66–1.09) | 1.00 (0.73–1.26) | 1.07 (0.56–2.06) |
| Ehime | 1.02 (0.83–1.24) | 0.97 (0.74–1.28) | 1.14 (0.67–1.96) |
| Kochi | 0.97 (0.76–1.24) | 0.82 (0.56–1.19) | 0.82 (0.40–1.71) |
| Fukuoka | 1.36 (1.21–1.54) | 1.84 (1.58–2.15) | 1.95 (1.39–2.73) |
| Saga | 1.15 (0.92–1.45) | 1.28 (0.93–1.76) | 2.86 (1.70–4.81) |
| Nagasaki | 0.87 (0.70–1.07) | 0.92 (0.69–1.25) | 1.12 (0.63–1.98) |
| Kumamoto | 1.07 (0.89–1.29) | 0.97 (0.74–1.28) | 1.56 (0.95–2.58) |
| Oita | 1.18 (0.95–1.47) | 1.45 (1.09–1.94) | 1.69 (0.99–2.89) |
| Miyazaki | 1.04 (0.84–1.28) | 0.99 (0.73–1.36) | 0.67 (0.33–1.35) |
| Kagoshima | 1.09 (0.90–1.32) | 1.10 (0.85–1.41) | 1.41 (0.87–2.29) |
| Okinawa | 1.20 (1.00–1.43) | 2.06 (1.62–2.62) | 4.66 (3.28–6.61) |

Prefecture-specific random-effect odds ratios (ORs) and 95% confidence intervals (CIs) for favorable neurological outcomes were derived from mixed-effects logistic regression models. Values greater than 1 indicate better-than-average outcomes after adjusting for covariates, whereas values less than 1 indicate worse-than-average outcomes.

**Abbreviation:** PEA, pulseless electrical activity
